# The long-term cost-effectiveness of oral semaglutide versus empagliflozin and dulaglutide in Portugal

**DOI:** 10.1186/s13098-022-00801-4

**Published:** 2022-02-14

**Authors:** Samuel J. P. Malkin, Davide Carvalho, Catarina Costa, Vasco Conde, Barnaby Hunt

**Affiliations:** 1 Ossian Health Economics and Communications GmbH, Bäumleingasse 20, 4051 Basel, Switzerland; 2 Department of Endocrinology, Diabetes and Metabolism, Centro Hospitalar Universitário de S João, Faculty of Medicine and Instituto de Investigação e Inovação em Saúde, Universidade do Porto, Porto, Portugal; 3 Novo Nordisk Portugal, Lda, Paço de Arcos, Portugal

**Keywords:** Costs and cost analysis, Cost-effectiveness, Diabetes mellitus, Dulaglutide, Empagliflozin, GLP-1 receptor agonist, Oral semaglutide, Portugal

## Abstract

**Background:**

Oral semaglutide is a novel glucagon-like peptide-1 (GLP-1) analog that has been associated with improvements in glycated hemoglobin (HbA1c) and body weight versus sodium-glucose cotransporter-2 inhibitor empagliflozin and injectable GLP-1 receptor agonist dulaglutide in the PIONEER 2 clinical trial and in a recent network meta-analysis (NMA), respectively. The aim of the present study was to evaluate the long-term cost-effectiveness of oral semaglutide 14 mg versus empagliflozin 25 mg and dulaglutide 1.5 mg for the treatment of type 2 diabetes from a healthcare payer perspective in Portugal.

**Methods:**

In two separate analyses, outcomes were projected over patients’ lifetimes using the IQVIA CORE Diabetes Model (v9.0), discounted at 4% per annum. Clinical data were sourced from the PIONEER 2 trial and the NMA for the comparisons versus empagliflozin and dulaglutide, respectively. Patients were assumed to receive initial therapies until HbA1c exceeded 7.5%, then treatment-intensified to solely basal insulin therapy. Costs were accounted from a National Healthcare Service perspective in Portugal and expressed in 2021 euros (EUR). Utilities were taken from published sources.

**Results:**

Oral semaglutide 14 mg was associated with improvements in life expectancy of 0.10 and 0.03 years, and quality-adjusted life expectancy of 0.11 and 0.03 quality-adjusted life years (QALYs), versus empagliflozin 25 mg and dulaglutide 1.5 mg, respectively. Improved clinical outcomes were due to a reduced cumulative incidence and increased time to onset of diabetes-related complications with oral semaglutide. Total costs were projected to be EUR 2548 and EUR 814 higher with oral semaglutide versus empagliflozin and dulaglutide, with higher acquisition costs partially offset by cost savings from avoidance of diabetes-related complications. Oral semaglutide 14 mg was therefore associated with incremental cost-effectiveness ratios of EUR 23,571 and EUR 23,927 per QALY gained versus empagliflozin 25 mg and dulaglutide 1.5 mg, respectively.

**Conclusions:**

Based on a willingness-to-pay threshold of EUR 30,000 per QALY gained, oral semaglutide 14 mg was considered cost-effective versus empagliflozin 25 mg and dulaglutide 1.5 mg for the treatment of type 2 diabetes in Portugal.

## Background

Diabetes mellitus is one of the most common endocrine disorders in the world, affecting more than 450 million people in 2019 [1]. In Portugal, diabetes was estimated to affect 13.6% of the population (7.7 million people) in 2018, with type 2 diabetes constituting 90% of cases [1, 2]. Diabetes-related healthcare expenditure in Portugal was estimated to exceed EUR 1.3 billion in 2018 and EUR 1.6 billion in 2019, equating to approximately 7–8% of the total healthcare budget and 0.6–0.8% of Portuguese gross domestic product [1, 2]. The majority of expenditure is associated with the treatment of long-term diabetes-related complications, and landmark studies have indicated that the incidence of these complications can be reduced by improving glycemic control, lowering blood pressure and reducing body weight [3–6]. Improving diabetes care can therefore improve patient outcomes while offering value for money for the healthcare payer. As healthcare systems come under increasing strain worldwide, demonstrating both the effectiveness and cost-effectiveness of novel interventions is becoming increasingly important.

Modern therapies for the treatment of type 2 diabetes, such as glucagon-like peptide-1 (GLP-1) receptor agonists and sodium-glucose cotransporter-2 (SGLT-2) inhibitors, have consistently demonstrated efficacy in terms of reducing glycated hemoglobin (HbA1c) levels and body weight, while offering minimal risk of hypoglycemia. Indeed, recent updated guidance published by the European Association for the Study of Diabetes (EASD), followed in Portugal, indicated GLP-1 receptor agonists or SGLT-2 inhibitors as second-line therapies (following metformin) in patients with type 2 diabetes with high risk of atherosclerotic cardiovascular disease, heart failure, or chronic kidney disease [7, 8]. These guidelines also emphasize the importance of a holistic approach to diabetes care, rather than a sole focus on glycemic control [7, 8]. However, while SGLT-2 inhibitors are an orally administered class of medications, GLP-1 receptor agonists were, until recently, only available in injectable formulations, potentially limiting uptake in populations averse to injectable therapy. The development of the efficacious semaglutide molecule into a once-daily, orally administered tablet, using absorption enhancer sodium *N*‐(8‐[2‐hydroxybenzoyl] amino) caprylate to facilitate absorption across the gastric mucosa, could therefore overcome these concerns.

Oral semaglutide 14 mg has been associated with improvements in HbA1c and body weight versus SGLT-2 inhibitor empagliflozin 25 mg in the PIONEER 2 clinical trial, and versus injectable GLP-1 receptor agonist dulaglutide 1.5 mg in a published network meta-analysis (NMA) [9, 10]. Moreover, oral semaglutide, empagliflozin and dulaglutide have been associated with cardiovascular benefits in the PIONEER 6, EMPA-REG OUTCOME and REWIND trials, respectively [11–13]. The cost-effectiveness of oral semaglutide versus empagliflozin, sitagliptin and liraglutide has been previously evaluated in both the UK and the Netherlands, but no study to date has evaluated the cost-effectiveness of oral semaglutide versus dulaglutide in a European setting [14, 15]. Moreover, healthcare systems and reimbursement strategies between countries can vary drastically, with differences in country-specific mortality, the costs of medications and the costs of treating diabetes-related complications all influencing cost-effectiveness outcomes. As both empagliflozin and dulaglutide are widely used in Portugal, comparison of oral semaglutide with these medications in terms of elucidating efficacy and cost-effectiveness over the long term can offer valuable information to physicians and healthcare payers.

The present analysis aimed to evaluate the long-term cost-effectiveness of oral semaglutide 14 mg versus empagliflozin 25 mg and dulaglutide 1.5 mg for the treatment of people with type 2 diabetes with inadequate glycemic control on 1–2 oral antidiabetic medications (OADs) in Portugal, in two separate analyses based on the results of PIONEER 2 and a recent NMA, respectively.

## Methods

### Modeling approach

In line with methodological guidelines for the economic evaluation of health technologies in Portugal, long-term outcomes were projected over patients’ lifetimes using the IQVIA CORE Diabetes Model (version 9.0) [16]. The features and capabilities of the model have been previously published [17]. Briefly, the model is a non-product-specific, validated and widely used diabetes policy analysis tool based on a series of inter-dependent sub-models that simulate diabetes-related complications, projecting the natural course of diabetes and associated clinical and cost outcomes [18, 19]. Relevant model outputs include life expectancy, quality-adjusted life expectancy (expressed in quality-adjusted life years [QALYs]), direct costs, time to onset of diabetes-related complications, cost-effectiveness scatterplots and acceptability curves, and incremental cost-effectiveness ratios (ICERs).

Projected cost and clinical outcomes were discounted at 4% annually, in line with modeling guidelines for Portugal [16]. All analyses were performed over a time horizon of 50 years, capturing patients’ lifetimes. The base case and sensitivity analyses were performed with a first-order Monte Carlo approach, while probabilistic analysis was performed with a second-order Monte Carlo approach. Model outcomes were predicted using the United Kingdom Prospective Diabetes Study (UKPDS) 68 risk equations, with a sensitivity analysis performed using the UKPDS 82 risk equations (as recommended by the model proprietors) [19]. The model captured mortality due to diabetes-related complications and background mortality based on Portugal-specific life tables published by the World Health Organization (WHO) [20].

### Clinical data and parameter progression

Separate analyses were performed to compare oral semaglutide with empagliflozin and oral semaglutide with dulaglutide, using different data sets. Baseline cohort characteristics for the comparison of oral semaglutide with empagliflozin were based on the PIONEER 2 trial, with corresponding treatment effects (changes from baseline in HbA1c, body mass index [BMI], blood pressure, serum lipids and estimated glomerular filtration rate [eGFR]) and hypoglycemic event rates sourced directly from the trial (Tables 1 and 2). PIONEER 2 enrolled people with type 2 diabetes with an HbA1c level of 7.0–10.5% receiving metformin at baseline and used two estimands to address two different efficacy questions [21, 22]. The treatment policy estimand included all study participants randomly assigned to each treatment, using data regardless of discontinuation of study medications and/or use of additional blood glucose lowering medications during the trial (thereby reflecting the intention-to-treat principle). In contrast, treatment effects evaluated by the trial product estimand assumed that patients received the study drug for the duration of the trial and did not receive any additional blood glucose lowering medications (thereby aiming to reflect the effects of the study medications without the confounding effects of rescue medication or any other changes in glucose-lowering medication) [21]. Data evaluated by the trial product estimand are therefore more appropriate for use in cost-effectiveness analyses, as the confounding clinical and cost impacts of additional medications (for example, additional reductions in HbA1c and the acquisition costs of these medications) are excluded, allowing a more direct comparison of the two evaluated interventions. Analyses based on PIONEER 2 were therefore performed using 52-week data evaluated by the trial product estimand.

**Table 1:** Baseline cohort characteristics

Characteristic	PIONEER 2 (oral semaglutide versus empagliflozin)	PIONEER 3 (oral semaglutide versus dulaglutide)
Start age (years)	57.63 (9.94)	57.86 (9.87)
Duration of diabetes (years)	7.00 (6.09)*	9.00 (5.98)*
Percentage male (%)	50.55	52.82
HbA1c (%)	8.14 (0.94)	8.31 (0.92)
Systolic blood pressure (mmHg)	132.15 (14.69)	133.83 (15.41)
BMI (kg/m^2^)	32.82 (6.11)	32.49 (6.41)
Percentage smokers (%)	14.62	14.17
Cigarettes per day	13.00*,^†^	
Alcohol consumption (oz/week)	7.97^†^	

BMI: body mass index; HbA1c: glycated hemoglobin

*Rounded values, as the IQVIA CORE Diabetes Model only accepts integers for these parameters

^†^Based on Portugal-specific data as not available from the PIONEER trial program

**Table 2:** Treatment effects applied in the analyses

Parameter	PIONEER 2		NMA	
	Oral semaglutide 14 mg	Empagliflozin 25 mg	Oral semaglutide 14 mg	Dulaglutide 1.5 mg
*Physiological parameters (applied in the first year of the analysis)*				
HbA1c (%)	− 1.30 (0.05)*	− 0.79 (0.05)	− 1.50 (0.13)	− 1.29 (0.11)
Systolic blood pressure (mmHg)	− 4.85 (0.65)	− 4.34 (0.63)	− 3.09 (1.13)	− 3.57 (1.08)
Total cholesterol (mg/dL)	− 5.08 (1.62)*	4.74 (1.57)	0 (0)^†^	0 (0)^†^
HDL cholesterol (mg/dL)	0.73 (0.35)*	3.11 (0.34)	0 (0)^†^	0 (0)^†^
BMI (kg/m^2^)	− 1.73 (0.10)*	− 1.37 (0.09)	− 1.50 (0.18)*	− 0.73 (0.17)
*Adverse event rates (applied while patients received initial therapies)*				
Non-severe hypoglycemia event rate (events per 100 patient years)	2.25	1.90	0.00^†^	0.00^†^
Severe hypoglycemia event rate (events per 100 patient years)	0.25	0.24	0.00^†^	0.00^†^
Proportion of nocturnal non-severe hypoglycemic events	0.11	0.13	0.00^†^	0.00^†^
Proportion of nocturnal severe hypoglycemic events	0.00	0.00	0.00^†^	0.00^†^

BMI: body mass index; HbA1c: glycated hemoglobin; HDL: high-density lipoprotein; NMA: network meta-analysis. Values are means (standard errors)

*Statistically significant difference at 95% confidence level for oral semaglutide versus the comparator

^†^Not included in the NMA and therefore assumed to be zero

For the comparison of oral semaglutide with dulaglutide, baseline cohort characteristics were sourced from PIONEER 3, as this trial was used to inform the oral semaglutide arm of the NMA (Table 1) [10, 23]. PIONEER 3 enrolled people with type 2 diabetes with an HbA1c level of 7.0–10.5% receiving metformin with or without a sulfonylurea at baseline [23]. The PIONEER 4 randomized controlled trial was also included in the NMA, and therefore represented an option to inform the baseline cohort characteristics [24]. However, the higher baseline HbA1c in the PIONEER 3 trial (8.31% in PIONEER 3 compared with 7.96% in PIONEER 4) was felt to be more aligned with target population in Portugal, and to correlate with the large reductions in HbA1c observed in the NMA with oral semaglutide 14 mg, and therefore the PIONEER 3 data were used in the base case analysis. Changes in physiological parameters were applied from the NMA, which assessed changes from baseline in HbA1c, BMI and blood pressure (Table 2) [10]. Changes in other physiological parameters, such as serum lipids and eGFR, and hypoglycemic event rates were assumed to be zero to avoid any assumptions influencing cost-effectiveness outcomes.

For both comparisons, all treatment effects (including those that were non-statistically significant) between the treatment arms were applied, in line with health economic guidelines [25]. Alcohol and tobacco consumption for both comparisons were sourced from Portugal-specific data for the general population, as these data were not collected in the PIONEER 2 or 3 clinical trials [26, 27].

Treatment effects from either PIONEER 2 or the NMA were applied in the first year of the analysis, after which HbA1c was modeled to follow the UKPDS progression equation, which resulted in HbA1c increasing over time and differences between the treatment arms gradually reducing. After HbA1c exceeded 7.5%, patients were assumed to discontinue oral semaglutide, empagliflozin or dulaglutide therapy and treatment-intensify to insulin Abasaglar^®^, with a corresponding, calculated reduction in HbA1c applied based on the “Core” multivariate equations for an insulin-naïve population estimated by Willis et al. [28]. This approach meant that patients received initial medications for different lengths of time (3 years in the oral semaglutide and dulaglutide arms and 2 years in the empagliflozin arm), reflecting differences in glycemic control with each intervention. Treatment effects for BMI were assumed to persist while patients received initial therapies, and reverted to baseline on intensification to basal insulin. Blood pressure and serum lipids were modeled to follow the natural progression algorithms built into the IQVIA CORE Diabetes Model, based on UKPDS and Framingham data, respectively [17]. Hypoglycemia rates after patients intensified to basal insulin were based on published data, with non-severe and severe hypoglycemic events projected to increase to 4.08 and 0.10 events per patient per year, respectively [29]. The HbA1c progression and treatment intensification approach used in the present study has been used in several previously published cost-effectiveness analyses [14, 15, 30].

### Cost data

Costs were accounted from the perspective of the National Health Service (NHS) in Portugal and expressed in 2021 euros (EUR). In line with modeling guidelines for Portugal, captured costs included all costs falling within the NHS budget [16]. Costs included pharmacy costs, costs of treating diabetes-related complications, and patient management costs. Unit costs of diabetes medications were based on the pharmacy selling price (PSP) including value-added tax (VAT) from Spring 2021 and captured the appropriate reimbursement levels. These unit costs were used to calculate annual costs of treatment for each arm, based on the resource use from the PIONEER 2 and 3 clinical trials for comparisons with empagliflozin and dulaglutide, respectively. No self-monitoring of blood glucose (SMBG) use was associated with oral semaglutide or comparator treatment, and no needle use was required, as both oral semaglutide and empagliflozin are administered orally, and needles are included in the dulaglutide packs. Following intensification to basal insulin therapy, patients were assumed to require 40 IU of insulin Abasaglar^®^ (based on the defined daily dose), one needle and one SMBG test per day [31]. Costs of treating diabetes-related complications and patient management costs were sourced from NHS tariffs where possible, with peer-reviewed publications and expert advice used to fill data gaps.

### Utilities

Health-state utilities and event-based disutilities associated with diabetes-related complications were based on a 2014 literature review conducted by Beaudet et al*.*, with hypoglycemia disutilities coming from a 2013 publication by Evans et al. (published after the literature searches by Beaudet et al. had been completed) [32, 33]. The review captured utility data from a wide range of published sources (Table 3) [34–39]. Utilities elicited using the EQ-5D were preferentially selected by Beaudet et al., in line with guidance on economic evaluation in the Portuguese setting [16].

**Table 3:** Health-state utilities and event-based disutilities applied in the analyses

Complication	Utility	References
Patient with type 2 diabetes baseline (no complications)	0.785	[34]
Myocardial infarction event	− 0.055	[34]
Post-myocardial infarction	0.730	[34]
Angina	0.695	[34]
Congestive heart failure	0.677	[34]
Stroke event	− 0.164	[34]
Post-stroke	0.621	[34]
Peripheral vascular disease	0.724	[35]
Microalbuminuria	0.785	[34]
Gross proteinuria	0.737	[35]
Hemodialysis	0.621	[36]
Peritoneal dialysis	0.581	[36]
Renal transplant	0.762	[37]
Background diabetic retinopathy	0.745	[38]
Background diabetic retinopathy (wrongly treated)	0.745	[38]
Proliferative diabetic retinopathy (laser treated)	0.715	[38]
Proliferative diabetic retinopathy (no laser treatment)	0.715	[38]
Macular edema	0.745	[38]
Severe vision loss	0.711	[34]
Cataract	0.769	[39]
Neuropathy	0.701	[35]
Healed ulcer	0.785	[34]
Active ulcer	0.615	[35]
Amputation event	− 0.280	[34]
Post-amputation	0.505	[34]
Non-severe hypoglycemic event (daytime)	− 0.004	[33]
Non-severe hypoglycemic event (nocturnal)	− 0.007	[33]
Severe hypoglycemic event (daytime)	− 0.057	[33]
Severe hypoglycemic event (nocturnal)	− 0.062	[33]
Each unit of BMI over 25 kg/m^2^	− 0.0061	[35]

BMI: body mass index

Health-state utilities are calculated as the baseline utility for type 2 diabetes with no complications plus the corresponding event-based disutilities sourced from the literature review conducted by Beaudet et al. [32]

### Key drivers of outcomes

A series of analyses were performed to evaluate the key drivers behind cost-effectiveness outcomes. Separate analyses applied the differences in HbA1c, blood pressure, serum lipids, BMI and hypoglycemia in the oral semaglutide arm in turn, with all other parameters set to the values observed in the comparator arm. For the comparison with dulaglutide, only the differences in HbA1c, blood pressure and BMI were tested, as these were the only outcomes included in the NMA [10].

### Sensitivity analyses

Projection of long-term outcomes from short-term data is inherently associated with uncertainty. To examine the impact of alternative model inputs on cost-effectiveness outcomes, a series of sensitivity analyses were performed to assess the robustness of the model results. These included: applying shorter time horizons of 35, 20 and 10 years in separate analyses (for which it should be noted that not all clinical and cost outcomes were captured, as not all simulated patients had died at the end of the analyses); applying symmetrical discount rates of 0% and 3% in separate analyses; maintaining changes from baseline in BMI in both arms for the duration of the analysis; applying no changes in HbA1c on treatment intensification to basal insulin; applying the upper and lower 95% confidence intervals of the estimated treatment differences in HbA1c and BMI in separate analyses; applying a linear HbA1c progression with treatment switching after 3 years, and HbA1c brought to 7.0% on intensification; varying the costs of treating diabetes-related complications by ± 10%; applying the UKPDS 82 risk equations to predict model outcomes; applying an alternative disutility for changes in BMI; applying alternative hypoglycemia disutilities, including a diminishing hypoglycemia disutility model; reducing the price of the comparator by 10%; and performing the analysis with a second-order, probabilistic approach [39–41].

### Compliance with ethics guidelines

This article is based on previously conducted studies and does not contain any studies with human participants or animals performed by any of the authors.

## Results

### Base case analysis

Long-term projections in patients with inadequate glycemic control on 1–2 OADs indicated that oral semaglutide 14 mg was associated with improvements in life expectancy of 0.10 and 0.03 years, and quality-adjusted life expectancy of 0.11 and 0.03 QALYs, versus empagliflozin 25 mg (based on PIONEER 2) and dulaglutide 1.5 mg (based on an NMA), respectively (Table 4). Clinical benefits were due to a reduced incidence and increased time to onset of diabetes-related complications over the course of the analysis.

**Table 4:** Base case analysis results

Health outcomes	PIONEER 2		
	Oral semaglutide 14 mg	Empagliflozin 25 mg	Difference
Discounted life expectancy (years)	12.47 (0.16)	12.37 (0.17)	+ 0.10
Discounted quality-adjusted life expectancy (QALYs)	8.16 (0.11)	8.05 (0.11)	+ 0.11
Discounted direct costs (EUR)	25,930 (532)	23,382 (542)	+ 2548
ICER based on direct costs	EUR 23,571 per QALY gained		

Health outcomes	NMA		
	Oral semaglutide 14 mg	Dulaglutide 1.5 mg	Difference
Discounted life expectancy (years)	12.02 (0.16)	11.99 (0.18)	+ 0.03
Discounted quality-adjusted life expectancy (QALYs)	7.78 (0.11)	7.75 (0.12)	+ 0.03
Discounted direct costs (EUR)	26,626 (612)	25,812 (619)	+ 814
ICER based on direct costs	EUR 23,927 per QALY gained		

Values are means (standard deviations). EUR: euros; ICER: incremental cost-effectiveness ratio; NMA: network meta-analysis; QALYs: quality-adjusted life years

**Fig. 1:**
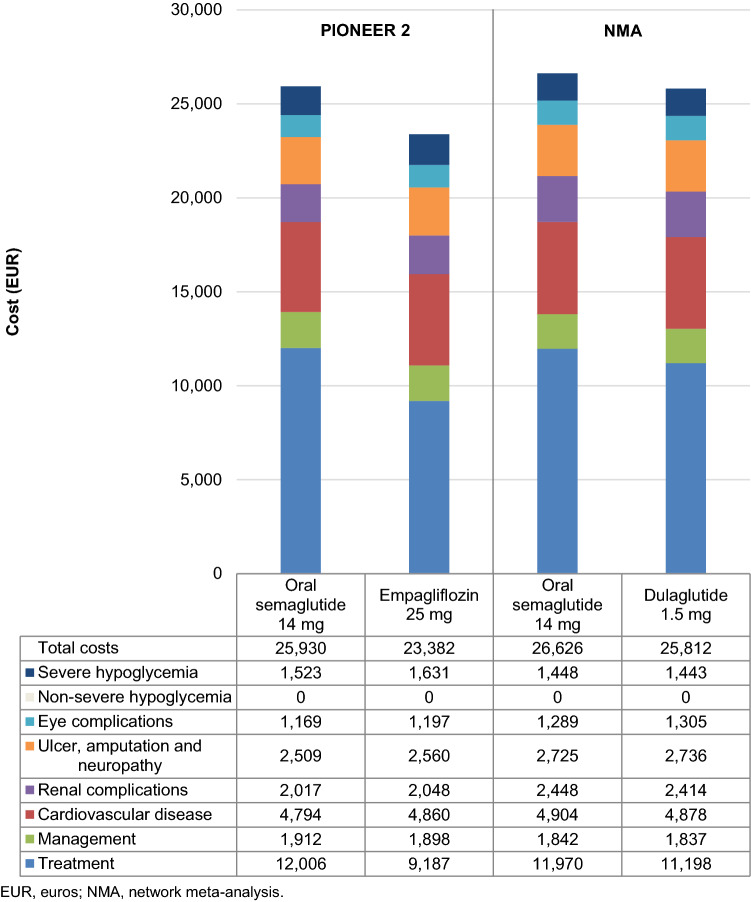
Total direct costs over patients’ lifetimes

Total direct costs over patients’ lifetimes were projected to be increased with oral semaglutide 14 mg, by EUR 2548 versus empagliflozin 25 mg and by EUR 814 versus dulaglutide 1.5 mg (Fig. 1). Increased costs were due to the higher acquisition cost of oral semaglutide, but these were partially offset by cost savings from avoidance of diabetes-related complications in the oral semaglutide arm (most notably severe hypoglycemia versus empagliflozin [mean cost savings of EUR 108 per patient] and ophthalmic complications versus dulaglutide [mean cost savings of EUR 16 per patient]). Small increases in the costs of treating cardiovascular and renal complications with oral semaglutide versus dulaglutide were due to increased survival of patients over the long term, who required further treatment of costly complications such as peripheral vascular disease, myocardial infarction and end-stage renal disease.

Estimation of long-term outcomes indicated that oral semaglutide was associated with improved clinical outcomes and increased costs versus empagliflozin and dulaglutide. Oral semaglutide 14 mg was therefore associated with ICERs of EUR 23,571 per QALY gained versus empagliflozin 25 mg and EUR 23,927 per QALY gained versus dulaglutide 1.5 mg from the perspective of the NHS in Portugal. Based on a willingness-to-pay threshold of EUR 30,000 per QALY gained, oral semaglutide 14 mg was considered cost-effective versus empagliflozin 25 mg and dulaglutide 1.5 mg for the treatment of type 2 diabetes in Portugal.

### Key drivers of clinical benefits

Applying each of the differences in HbA1c, blood pressure, serum lipids, BMI and hypoglycemia in turn indicated that change in HbA1c was the biggest driver of clinical benefits for oral semaglutide versus empagliflozin, and change in BMI was the biggest driver of clinical benefits for oral semaglutide versus dulaglutide, with quality-adjusted life expectancy improvements of 0.09 and 0.02 QALYs, respectively, when only these differences between the treatment arms were applied. In the comparison with empagliflozin, differences in blood pressure, serum lipids, BMI and hypoglycemia made negligible contributions to the clinical benefits with oral semaglutide. In the comparison with dulaglutide, differences in HbA1c made small contributions to improved outcomes with oral semaglutide, while application of only differences in blood pressure led to marginally improved outcomes with dulaglutide.

### Sensitivity analyses

Sensitivity analyses showed that the results of the base case findings were robust to changes in input parameters and assumptions, with ICERs in the majority of analyses remaining under a willingness-to-pay threshold of EUR 30,000 per QALY gained (Table 5). In the comparison of oral semaglutide with empagliflozin, the largest increase in the ICER was observed when applying a 10-year time horizon, while the largest decrease was observed when applying discount rates of 0%. Both of these analyses demonstrated that oral semaglutide was primarily associated with long-term benefits versus empagliflozin, and reinforce the importance of projecting outcomes over patients’ lifetimes. In the comparison of oral semaglutide with dulaglutide, the largest increase in the ICER was observed when applying only differences in treatment effects that were statistically significant in the NMA, where the only difference between the treatment arms was the change in BMI. Nonetheless, oral semaglutide was still associated with improved clinical outcomes over the long term. The largest decrease in the ICER was observed when maintaining the treatment effects in BMI for patients’ lifetimes, with the greater reductions in BMI associated with oral semaglutide yielding improved life expectancy and quality-adjusted life expectancy over the long term.

**Table 5:** Sensitivity analysis results

Analysis	Oral semaglutide 14 mg versus empagliflozin 25 mg (PIONEER 2)			Oral semaglutide 14 mg versus dulaglutide 1.5 mg (NMA)		
	Δ discounted quality-adjusted life expectancy (QALYs)	Δ discounted direct costs (EUR)	ICER (EUR per QALY gained)	Δ discounted quality-adjusted life expectancy (QALYs)	Δ discounted direct costs (EUR)	ICER (EUR per QALY gained)
Base case	+ 0.11	+ 2548	23,571	+ 0.03	+ 814	23,927
Statistically significant differences only	+ 0.10	+ 2512	25,456	+ 0.02	+ 828	36,478
35-year time horizon	+ 0.09	+ 2506	27,295	+ 0.04	+ 714	18,686
20-year time horizon	+ 0.08	+ 2452	32,014	+ 0.03	+ 704	24,178
10-year time horizon	+ 0.05	+ 2517	53,097	+ 0.02	+ 697	32,264
0% discount rates	+ 0.19	+ 2882	15,267	+ 0.06	+ 1068	18,219
3% discount rates	+ 0.12	+ 2610	21,357	+ 0.04	+ 856	22,419
BMI treatment effects maintained for patient lifetimes	+ 0.11	+ 2515	22,438	+ 0.08	+ 772	10,235
UKPDS HbA1c progression with no changes on treatment switch	+ 0.08	+ 4029	48,721	+ 0.02	+ 654	28,071
Upper 95% CI of HbA1c estimated treatment difference	+ 0.11	+ 2426	21,973	+ 0.05	+ 733	13,518
Lower 95% CI of HbA1c estimated treatment difference	+ 0.09	+ 2570	27,199	+ 0.03	+ 804	24,210
Upper 95% CI of BMI estimated treatment difference	+ 0.12	+ 2570	21,706	+ 0.04	+ 769	18,444
Lower 95% CI of BMI estimated treatment difference	+ 0.10	+ 2539	26,099	+ 0.02	+ 793	35,259
Treatment switching at 3 years with linear HbA1c progression	+ 0.07	+ 2753	41,086	+ 0.04	+ 771	18,760
Costs of complications+ 10%	+ 0.11	+ 2521	23,320	+ 0.03	+ 818	24,050
Costs of complications -10%	+ 0.11	+ 2575	23,821	+ 0.03	+ 809	23,805
UKPDS 82 risk equations applied	+ 0.08	+ 2395	29,931	+ 0.03	+ 705	28,678
Lee et al*.* BMI disutility applied	+ 0.11	+ 2548	22,410	+ 0.04	+ 817	20,961
Lauridsen et al*.* diminishing hypoglycemia model applied	+ 0.11	+ 2548	22,791	+ 0.03	+ 814	23,998
Currie et al*.* hypoglycemia disutilities applied	+ 0.10	+ 2548	24,642	+ 0.03	+ 814	23,787
Comparator price reduced by 10%	+ 0.11	+ 2644	24,457	+ 0.03	+ 1161	34,143

Δ: difference in; BMI: body mass index; CI: confidence interval; EUR: euros; HbA1c: glycated hemoglobin; ICER: incremental cost-effectiveness ratio; NMA: network meta-analysis; QALYs: quality-adjusted life years; UKPDS: United Kingdom Prospective Diabetes Study

**Fig. 2:**
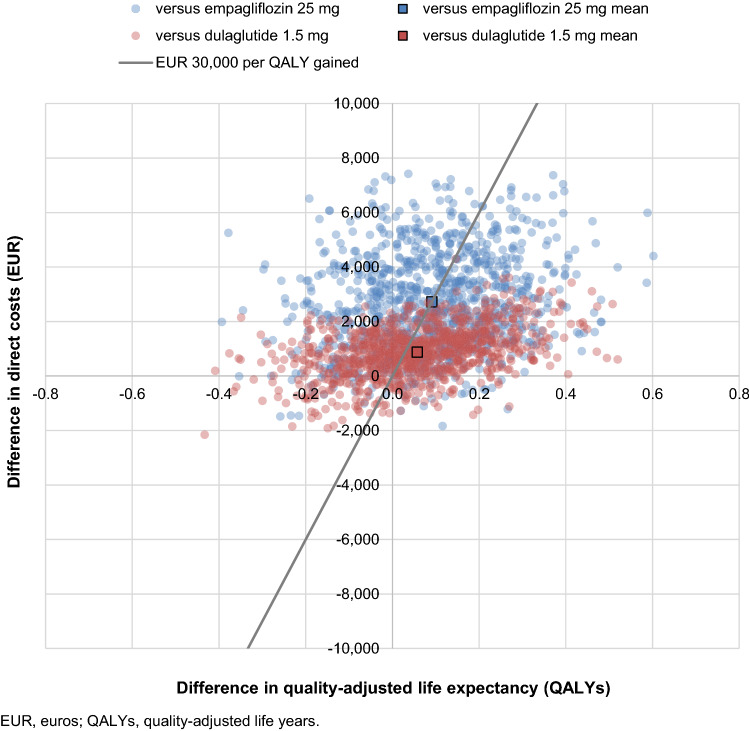
Probabilistic sensitivity analysis scatterplot

Probabilistic sensitivity analysis with sampling around cohort characteristics, treatment effects, complication costs and utilities yielded similar mean results to the base case analyses, but with increased measures of variance around outcomes. Oral semaglutide was projected to improve quality-adjusted life expectancy by 0.09 and 0.06 QALYs, and increase costs by EUR 2720 and EUR 877, versus empagliflozin and dulaglutide, respectively (Fig. 2). Oral semaglutide 14 mg was therefore associated with ICERs of EUR 29,920 per QALY gained versus empagliflozin 25 mg and EUR 15,258 per QALY gained versus dulaglutide 1.5 mg in the probabilistic analysis. Based on these analyses, the probabilities of oral semaglutide being cost-effective at a willingness-to-pay threshold of EUR 30,000 per QALY gained were estimated to be 51.5% and 58.3%, respectively.

## Discussion

The present analysis has demonstrated that treatment with oral semaglutide 14 mg is likely to lead to improved clinical outcomes versus treatment with empagliflozin 25 mg or dulaglutide 1.5 mg for people with type 2 diabetes with inadequate glycemic control on 1–2 OADs in Portugal. Although oral semaglutide was projected to increase costs over the long term, these were partially offset by cost savings from avoidance of diabetes-related complications, and ICERs in the base case analyses and in the majority of wide-ranging sensitivity analyses were estimated to be under the commonly used willingness-to-pay threshold of EUR 30,000 per QALY gained in Portugal. Oral semaglutide 14 mg was therefore considered cost-effective versus empagliflozin 25 mg and dulaglutide 1.5 mg.

Development of the semaglutide molecule for oral administration has allowed the efficacy benefits of a GLP-1 receptor agonist to be combined with the ease-of-use of other modern, orally administered diabetes treatments, such as SGLT-2 inhibitors. Oral semaglutide therefore has the potential to avoid the burden associated with injectable therapy and overcome barriers leading to therapeutic inertia [42–45]. While GLP-1 receptor agonists and SGLT-2 inhibitors are both recommended as second-line therapies by the EASD (guidance that is followed in Portugal), offering the choice of an oral GLP-1 analog at this stage in the algorithm affords both patients and physicians more choice when developing an individualized diabetes treatment program [7, 8]. Moreover, the present analysis did not take into account any potential quality-of-life impact from the once-weekly injection of dulaglutide versus the once-daily oral administration of oral semaglutide, and this could therefore be seen as conservative from the perspective of oral semaglutide. Nonetheless, based on projections from the present analysis, use of oral semaglutide for the treatment of type 2 diabetes in place of empagliflozin or dulaglutide should deliver value for money for the NHS in Portugal. These results should help to influence health policy in Portugal and similar countries where GLP-1 receptor agonist and SGLT-2 inhibitor use is limited, despite their demonstrated efficacy [8, 46, 47].

Previous studies have evaluated the cost-effectiveness of oral semaglutide versus empagliflozin and dulaglutide, including studies in the UK, the Netherlands and Sweden that have produced consistent results, and studies in the US and Denmark that have produced conflicting outcomes, particularly for the comparison of oral semaglutide versus empagliflozin [14, 15, 48–50]. Studies published in the UK by Bain et al., in the Netherlands by Malkin et al. and in Sweden by Eliasson et al. projected oral semaglutide to be cost-effective, while studies in the US and Denmark, published by the Institute for Clinical and Economic Review and Ehlers et al., respectively, predicted that oral semaglutide was not cost-effective [14, 15, 48, 50]. However, the different methods used in these studies must be taken into account when comparing results. The UK and Netherlands studies utilized the same treatment intensification approach as the present study, whereby patients discontinued initial therapies after HbA1c exceeded 7.5%, but the US and Denmark studies applied a lifetime treatment duration for oral semaglutide and empagliflozin [14, 15, 48, 50]. Maintaining GLP-1 receptor agonist and SGLT-2 inhibitor therapy for patients’ lifetimes is at odds with published data from general practice in Europe, which indicated an average duration of GLP-1 receptor agonist treatment of just over 29 months and a discontinuation rate of 45% for SGLT-2 inhibitors at 2 years [51–54]. The approach used for HbA1c progression and treatment intensification in the present study was chosen to accurately reflect real-world clinical practice where treatments are continued while glycemic control is maintained, and intensified as type 2 diabetes naturally progresses over patients’ lifetimes [14, 15, 30]. Indeed, the lengths of time patients received oral semaglutide and dulaglutide in the present study (3 years) closely matched the published data from clinical practice in Europe, which indicated a substantially shorter treatment duration than the lifetime assumptions applied in the analyses published by the Institute for Clinical and Economic Review and Ehlers et al. [48, 50, 51].

That acknowledged, a potential criticism of the present analysis is that only one treatment pathway was tested, with initial therapies discontinued when HbA1c exceeded 7.5%—treatment algorithms that added basal insulin alongside oral semaglutide, empagliflozin or dulaglutide were not explored. However, there is a current paucity of evidence evaluating changes in physiological parameters when adding basal insulin therapy to GLP-1 receptor agonist or SGLT-2 inhibitor treatment, which severely hinders appropriate modeling of this algorithm over the long term. The present study therefore chose a simplified treatment pathway to best answer the research question of the cost-effectiveness of oral semaglutide versus empagliflozin and dulaglutide. Nevertheless, future research elucidating changes in physiological parameters with addition and discontinuation of a variety of diabetes therapies, and subsequent modeling studies incorporating these data, should be conducted.

The lack of incorporation of the cardiovascular benefits of oral semaglutide, empagliflozin and dulaglutide observed in cardiovascular outcomes trials (CVOTs) could also be seen as a limitation of the analysis. Oral semaglutide demonstrated reductions in cardiovascular mortality in PIONEER 6, empagliflozin was linked to reductions in composite cardiovascular endpoints and heart failure in EMPA-REG OUTCOME, and dulaglutide was associated with reductions in the composite endpoint of non-fatal myocardial infarction, non-fatal stroke or cardiovascular mortality in REWIND [11–13]. Outcomes from EMPA-REG OUTCOME were partially included in the Danish analysis by Ehlers et al., while outcomes from an NMA of CVOTs were utilized in the US analysis by the Institute for Clinical and Economic Review, but incorporation of CVOT data into long-term modeling analyses is challenging for a myriad of reasons. These include potentially inappropriate application of effects observed in populations with high cardiovascular risk in more general populations with type 2 diabetes, and risking double counting of benefits in modeling analyses where changes in surrogate physiological parameters that affect the incidence of cardiovascular complications (such as HbA1c, blood pressure, serum lipids and BMI) have already been applied (as the mechanisms of action behind the benefits observed in CVOTs are not yet understood) [55, 56]. Development of novel model frameworks and risk equations to allow application of these effects is therefore the focus of current research [55, 56].

The progression equation used for HbA1c in the present study, based on 20-year-old data from the UKPDS, could be seen as a limitation, as these data may no longer be representative of modern clinical practice. However, in absence of type 2 diabetes studies of similar length enrolling similar numbers of patients, these data still represent one of the most widely used and robust sources of evidence for informing HbA1c progression. Moreover, a key strength of the treatment switching approach used in the present study was the use of the multivariate equations published by Willis et al., which allowed application of specifically calculated, relevant reductions in HbA1c when intensifying to basal insulin [28]. This avoided the need for assumptions around HbA1c reductions on intensification to basal insulin that could artificially influence model outcomes.

The two analyses used clinical data from separate sources, with the PIONEER 2 trial used to inform the oral semaglutide versus empagliflozin analysis and an NMA used to inform the oral semaglutide versus dulaglutide analysis. Therefore, oral semaglutide was associated with different treatment effects in the two analyses. While this may limit the comparison across the analyses (i.e., no conclusions on the relative cost-effectiveness of dulaglutide versus empagliflozin can be drawn), this approach was chosen to ensure that the most robust source of clinical data was used in the two analyses.

The long-term cost-effectiveness of antidiabetic medications is particularly relevant in light of the COVID-19 pandemic. Patients with type 2 diabetes are at a higher risk of developing severe, COVID-19-related complications, and individuals experiencing diabetes-related complications that require hospitalization can be exposed to a higher risk of catching COVID-19 [57]. Moreover, improving patient-physician contact and interactions between specialists working in different disciplines, particularly via telephone or online, has been highlighted as a unique opportunity to improve diabetes care in the era of social distancing, isolation and quarantine [57, 58]. Providing clear information on the effectiveness and cost-effectiveness of medications can therefore help to improve communication around diabetes care in Portugal.

The use of NMA data in the analysis could be seen as a potential weakness. However, no head-to-head clinical trial has directly compared oral semaglutide 14 mg with dulaglutide 1.5 mg, and selection of the most appropriate comparators for the Portuguese setting was the first priority. Moreover, the use of evidence synthesis, using recommended methodologies, is becoming increasingly important and accepted for health technology assessment globally [59, 60]. A series of wide-ranging sensitivity analyses were also performed to examine the impact of changes to input parameters and assumptions, and this represents a key strength of the present study. In addition, the use of data from an NMA allowed the analysis to evaluate the cost-effectiveness of oral semaglutide versus dulaglutide, which adds new evidence to the literature alongside the previously published cost-effectiveness analyses of oral semaglutide [14, 15]. The NMA included several other comparators that were beyond the scope of the current analysis, but future studies could potentially evaluate the cost-effectiveness of oral semaglutide versus these comparators should they be relevant or of interest in other country settings.

The extrapolation of short-term clinical data to long-term outcomes is also inherently associated with uncertainty. However, this is an essential tenet of diabetes modeling over patients’ lifetimes, and represents arguably the best source of evidence to inform decision making in absence of long-term clinical trial data. Moreover, every effort was made to minimize uncertainty in the analysis, by using an extensively published and validated health economic model, by utilizing clinical input data from published studies and by performing a series of extensive sensitivity analyses that demonstrated the outcomes of the base case analyses were robust [9, 10, 17–19].

## Conclusions

Based on a willingness-to-pay threshold of EUR 30,000 per QALY gained, oral semaglutide 14 mg represents a cost-effective option versus empagliflozin 25 mg and dulaglutide 1.5 mg for the treatment of people with type 2 diabetes with inadequate glycemic control on 1–2 OADs in Portugal.

## Data Availability

The datasets used and analyzed during the current study are available from the corresponding author on reasonable request.

## Abbreviations

BMI: Body mass index
CVOT: Cardiovascular outcomes trial
EASD: European Association for the Study of Diabetes
eGFR: Estimated glomerular filtration rate
EUR: 2021 Euros
GLP-1: Glucagon-like peptide-1
HbA1c: Glycated hemoglobin
HDL: High-density lipoprotein
ICER: Incremental cost-effectiveness ratio
NHS: National Health Service
NMA: Network meta-analysis
OAD: Oral antidiabetic medication
PSP: Pharmacy selling price
QALY: Quality-adjusted life year
SGLT-2: Sodium-glucose cotransporter-2
SMBG: Self-monitoring of blood glucose
UKPDS: United Kingdom Prospective Diabetes Study
VAT: Value-added tax
WHO: World Health Organization
